# Monochromatic light increases anthocyanin content during fruit development in bilberry

**DOI:** 10.1186/s12870-014-0377-1

**Published:** 2014-12-16

**Authors:** Laura Zoratti, Marian Sarala, Elisabete Carvalho, Katja Karppinen, Stefan Martens, Lara Giongo, Hely Häggman, Laura Jaakola

**Affiliations:** Department of Biology, University of Oulu, PO Box 3000, FI-90014 Oulu, Finland; Plant Molecular Science, Centre for Systems and Synthetic Biology, Royal Holloway University of London, TW20 0EX Egham, UK; Fondazione Edmund Mach, Research and Innovation Center, via E. Mach 1, 38010S Michele all’Adige, TN Italy; Climate laboratory, Department of Arctic and Marine Biology, UiT the Arctic University of Norway, NO-9037 Tromsø, Norway; Norwegian Institute for Agricultural and Environmental Research, Bioforsk Nord Holt, Box 2284, NO-9269 Tromsø, Norway

**Keywords:** Light quality, *Vaccinium myrtillus* L, Flavonoids, Anthocyanins, Bilberry, Berries, UPLC**-**MS/MS

## Abstract

**Background:**

Light is one of the most significant environmental factors affecting to the accumulation of flavonoids in fruits. The composition of the light spectrum has been shown to affect the production of phenolic compounds during fruit ripening. However, specific information on the biosynthesis of flavonoids in fruits in response to different wavelengths of light is still scarce. In the present study bilberry (*Vaccinium myrtillus* L.) fruits, which are known to be rich with anthocyanin compounds, were illuminated with blue, red, far-red or white light during the berry ripening process. Following the illumination, the composition of anthocyanins and other phenolic compounds was analysed at the mature ripening stage of fruits.

**Results:**

All the three monochromatic light treatments had significant positive effect on the accumulation of total anthocyanins in ripe fruits compared to treatment with white light or plants kept in darkness. The elevated levels of anthocyanins were mainly due to a significant increase in the accumulation of delphinidin glycosides. A total of 33 anthocyanin compounds were detected in ripe bilberry fruits, of which six are novel in bilberry (cyanidin acetyl-3-*O*-galactose, malvidin acetyl-3-*O*-galactose, malvidin coumaroyl-3-*O*-galactose, malvidin coumaroyl-3-*O*-glucose, delphinidin coumaroyl-3-*O*-galactose, delphinidin coumaroyl-3-*O*-glucose).

**Conclusions:**

Our results indicate that the spectral composition of light during berry development has significant effect on the flavonoid composition of ripe bilberry fruits.

**Electronic supplementary material:**

The online version of this article (doi:10.1186/s12870-014-0377-1) contains supplementary material, which is available to authorized users.

## Background

Anthocyanins, a class of flavonoid compounds, are the main pigments found in many flowers and fruits, in which they act as insect and animal attractants and protect the plant from light oxidative stress [1]. Furthermore, these metabolites are powerful antioxidants and therefore shown to be beneficial for human health [2]. Several reports have focused on their effects in the prevention of neuronal and cardiovascular diseases, cancer and diabetes as well as in promoting human nutrition [2,3].

Bilberry (*Vaccinium myrtillus* L.) is among the most significant wild berry species in the Northern and Eastern Europe. Bilberry fruits are rich in phenolic acids, stilbenes and flavonoids, particularly in anthocyanins, which are estimated to represent nearly 90% of the total phenolics in these berries [4,5]. Anthocyanins are biosynthesized via the phenylpropanoid/flavonoid pathway consisting of a number of enzymatic steps that catalyze a sequential reaction leading to the production of different anthocyanidins including delphinidins (Dp), cyanidins (Cy), petunidins (Pt), peonidins (Pn) and malvidins (Mv) (Additional file 1). In bilberry fruits, the quantitative and qualitative composition of flavonoids is known to be strongly affected by the fruit developmental stage [6,7]. Bilberry fruits are known to accumulate high yields of various anthocyanins both in skin and flesh during the ripening period, although genetic and environmental factors are also reported to affect the final composition [8-10]. Two families of transcription factors, the bHLH and MYB proteins, are strongly associated in the regulation of the anthocyanin pathway [11,12]. The phenylpropanoid pathway responds to various environmental stimuli such as temperature, photoperiod, soil fertility [10,13,14] and light in particular [15,16].

Plants can sense multiple aspects of the light signals including light quantity (fluence), quality (wavelength), duration (photoperiod) and direction [17], which are perceived through at least four different families of photoreceptors, including phytochromes (red/far-red light receptors), cryptochromes and phototropins (blue light receptors) and UV-B photoreceptor (UVR8). These proteins perceive specific wavelengths of the visible light spectrum (380–740 nm) or the UV-light (280–315 nm) and transduce the signal to regulate photosynthesis, photomorphogenesis, phototropism, circadian rhythms as well as biosynthesis of secondary metabolites [18].

The induction of flavonoid and anthocyanin production by visible light has been extensively studied in several plant species, and it was found that the composition of light spectra regulated the biosynthesis of anthocyanins in *Arabidopsis* [19], cranberry (*Vaccinium macrocarpon* Ait.) [20], *Gerbera* [21], grape (*Vitis vinifera* L.) [22,23], lettuce (*Lactuca sativa* L.) [24], strawberry (*Fragaria x ananassa* -Weston- Duchesne ex Rozier) [25] and turnip (*Brassica napus* L.) [26]. A significant increase in the amount of phenolic compounds has been seen in bilberry plants grown under direct sunlight when compared to plants grown under forest canopy [9,15,27], but there is no information available on the effects of specific light wavelengths on their biosynthesis. Therefore, the aim of the present study was to analyze the influence of monochromatic wavelengths of the visible light spectrum on the production of phenolic compounds in bilberry fruit. Our particular interest was to study whether specific light wavelengths during berry development affect the biosynthesis and content of phenolic compounds. For this purpose, bilberry plants were illuminated with blue, red, far-red or white light during the berry ripening process and composition of the accumulated phenolic compounds was analyzed in ripe fruits. We also investigated the expression of key genes of bilberry flavonoid pathway in order to better understand the regulatory processes affecting biosynthesis of phenolic compounds during berry development.

## Results

### Characterization and quantification of phenolic compounds in ripe bilberry fruits

The phenolic compounds other than anthocyanins present in ripe bilberry fruits were analyzed by a UPLC-MS/MS method that has been earlier optimized for berry fruit species [28]. The phenolic compounds found in ripe bilberry fruits are listed in Table 1. The most abundant of those were hydroxycinnamic acids, namely chlorogenic acid and neochlorogenic acid. Naringenin (the precursor of flavonoid compounds) varied between 0.08 and 0.44 mg/100 g DW, and was present in much higher concentration in the glycosylated form (naringenin 7-*O*-glucoside) which, to our knowledge, is reported for the first time in bilberry in the present study. Also among stilbenes, (−)-astringin was detected in this study for the first time to our knowledge in bilberry fruits. The flavone luteolin 7-*O*-glucoside was found only in trace amounts.

**Table 1:** **Concentration of phenolic compounds (mg/100 g DW) detected in ripe bilberry fruits after monochromatic light treatment (n = 3)**

**Compound**	**Blue**			**Red**			**Far-red**			**White**			**Dark**		
	**Av.**	**SD**	**St.**	**Av.**	**SD**	**St.**	**Av.**	**SD**	**St.**	**Av.**	**SD**	**St.**	**Av.**	**SD**	**St.**
Neochlorogenic acid	80	35		129	29		105	27		8	27		96	21	
Chlorogenic acid	113	59		207	67		162	58		117	50		135	36	
**Total hydroxycinnamic acids**	193	94		96	24		168	55		133	40		100	24	
Naringenin	0.3	0.2		0.4	0.2		0.2	0.1		0.2	0.06		0.2	0.1	
Naringenin 7 glucoside**	82	26		70	9		69	7		50	17		68	29	
**Total flavanones**	83	26		70	9		69	7		50	17		68	29	
(−)-Astringin**	0.2	0.1		0.2	0.1		0.2	0.1		0.1	0.06		0.1	0.05	
**Total stilbenes**	0.2	0.1		0.2	0.1		0.2	0.1		0.1	0.06		0.1	0.05	
Kaempferol 3 rutinoside	4	1		4	2		5	1		4	2		4	3	
Quercetin 3 glu	5	3		3	1		4	2		4	2		4	2	
Quercetin 3 gal	9	3	b	17	2	a	15	10	a,b	17	4	a	21	12	a,b
Quercetin 3 glucuronide	35	1		46	9		28	9		42	5		36	3	
Syringetin 3 gal + glu	4	2		6	6		4	2		4	0.5		3	0.9	
Myricetin hexoses	7	2	b	12	1	a	12	1	a	5	1	b	8	3	b
**Total flavonols**	65	5		86	19		66	19		76	10		75	13	
Catechin	1	0.8		0.6	0.3		2	1		1	0.4		0.7	0.3	
Epicatechin	45	6		45	19		30	11		43	7		35	10	
Epigallocatechin	11	1		10	4		9	5		13	6		11	3	
Gallocatechin	15	1		14	5		12	6		17	7		14	4	
Procyanidin A2	0.25	0.07	a	0.08	0.06	b	0.09	0.03	b	0.25	0.10	a	0.23	0.18	a
Procyanidin B1	0.08	0.10	b	0.15	0.12	b	0.13	0.12	b	0.24	0.02	a	0.11	0.08	b
Procyanidin B2/B4	0	0		1.32	1.32		0.41	0.36		0.23	0.23		0.32	0.32	
Procyanidin B3	44	10		45	16		33	15		41	6		35	8	
**Total proanthocyanidins**	116	16		117	43		86	37		116	19		96	21	

glu = glucose, gal = galactose, Av. = average of three replicates, SD = standard deviation, St. = statistics.

The compounds marked with asterisk (**) are first time detected in bilberry fruits to present. Significant differences by Tukey HSD (P < 0.05) in response to the light treatments are marked by different letters for each compound and total amounts of compounds.

Ripe bilberries also contained flavonols, which included kaempferol 3-*O*-rutinoside, the quercetin derivatives (quercetin 3-*O*-glucose, quercetin 3-*O*-galactose, quercetin 3-*O*-glucuronide) and the myricetin derivatives (syringetin 3-*O*-glucose, syringetin 3-*O*-galactose and myricetin hexoses) in amounts comparable with earlier reports for bilberry [29].

The detected proanthocyanidins included monomers of catechin, epicatechin, epigallocatechin and gallocatechin. Among polymers, the most abundant was procyanidin B3 accompanied by lowers amounts of procyanidin A2, procyanidin B1, procyanidin B2 and/or B4 (which could not be separated using the present method [28]).

### Characterization and quantification of anthocyanins in ripe bilberry fruits

Anthocyanins are the most abundant class of flavonoids present in ripe bilberry fruits. The anthocyanin content in ripe bilberry fruits was analyzed by a UPLC-MS/MS method which had been earlier optimized for grapevine [30]. The method was slightly modified to allow the detection of anthocyanidin galactosides and arabinosides that have earlier been described for bilberry (see Methods).

The total amount of anthocyanins in ripe berries varied between 1860 to 3397 mg/100 g DW, which is comparable with the amounts reported earlier for bilberry [6,8]. Altogether 33 anthocyanins were detected (Table 2), including the known 15 anthocyanins; Dp’s, Cy’s, Pt’s, Pn’s and Mv’s combined with the sugars glucose, galactose and arabinose [8,31]. In addition, acetylated and *p*-coumaroyl-binded forms of anthocyanins, Pg’s and Cy 3-*O*-sambubioside compounds were found. To our knowledge, some of the acetylated (Cy acetyl 3-*O*-galactose and Mv acetyl 3-*O*-galactose) and coumaroylated compounds (Dp coumaroyl 3-*O*-glucose, Dp coumaroyl 3-*O*-galactose, Mv coumaroyl 3-*O*-glucose, Mv coumaroyl 3-*O*-galactose) that were detected in this study have not been previously reported in bilberry fruits. Acetylated compounds were present in low amounts, with an average concentration between 0.05 to 0.72 mg/100 g DW for the single compound detected (Table 2). The amount of *p*-coumaroylated anthocyanins was generally higher than the acetylated forms, even though the presence of these forms was more variable between the replicate plants. The contents ranged from the lowest of Mv coumaroyl 3-*O*-galactose to the highest of Pn and Mv coumaroyl 3-*O*-glucoside. However, the concentration of Pn and Mv coumaroyl 3-*O*-glucoside was in the same range with the known anthocyanins including Pt 3-*O*-glucoside, Pt 3-*O*-galactose, Mv 3-*O*-glucoside, Mv 3-*O*-galactose, Mv 3-*O*-arabinose, Pn 3-*O*-glucoside, Pn 3-*O*-galactose and Pn 3-*O*-arabinose (Table 2). The amounts of Pg derivatives were low in bilberry fruits, 0.36 mg/100 g of Pg 3-*O*-glucoside and 0.11 mg/100 g DW of Pg 3-*O*-galactose, while Pg 3-*O*-arabinose was not detected. The presence of Cy 3-*O*-sambubioside has also previously been reported in bilberry by Du et al. [32] in similar amounts that were found in our study.

**Table 2:** **Concentration of anthocyanin compounds (mg/100 g DW) detected in ripe bilberry fruits after monochromatic light treatment (n = 3)**

**Compound**	**Blue**			**Red**			**Far-red**			**White**			**Dark**		
	**Av.**	**SD**	**St.**	**Av.**	**SD**	**St.**	**Av.**	**SD**	**St.**	**Av.**	**SD**	**St.**	**Av.**	**SD**	**St.**
Cy acetyl 3 glu	0.59	0.88		0.49	0.64		0.33	0.16		0.54	0.38		0.74	0.73	
Pt acetyl 3 glu	0.10	0.09		0.12	0.10		0.08	0.01		0.07	0.06		0.26	0.45	
Pn acetyl 3 glu	0.18	0.07		0.11	0.09		0.21	0.10		0.39	0.24		1.12	1.42	
Mv acetyl 3 glu	0.35	0.30		0.43	0.50		1.04	0.53		0.75	0.24		4.54	4.54	
Cy acetyl 3 gal**	0.10	0.18		0.13	0.12		0.17	0.16		0.32	0.19		0.59	0.56	
Mv acetyl 3 gal**	0.17	0.11		0.17	0.05		0.34	0.16		0.26	0.12		0.52	0.52	
Dp acetyl 3 glu	0.07	0.06		0.05	0.09		0.00	0.00		0.03	0.05		0.00	0.00	
Cy coum 3 glu	10	3		8	7		11	4		9	5		24	27	
Dp coum 3 glu**	1.8	0.3		2.4	1.4		1.6	1.6		0.72	0.10		2	2	
Pn coum 3 glu	15	6		18	15		27	8		28	13		93	127	
Mv coum 3 glu**	24	9		31	12		41	5		24	2		121	181	
Cy coum 3 gal	3	1		4	0.6		4.5	0.7		5	2		7	6	
Dp coum 3 gal**	0.88	0.37		0.87	0.34		1.21	0.25		0.64	0.15		0.57	0.38	
Pn coum 3 gal	0.69	0.28		1.22	0.49		1.63	0.20		3.05	2.14		3.32	3.32	
Mv coum 3 gal**	0.22	0.21		0.52	0.44		0.97	0.22		0.49	0.41		1.96	1.96	
Cy sambubioside	2.4	0.48		2.3	1.9		1.46	0.49		1.91	1.21		2.36	1.99	
Cy 3 glu	600	174		470	314		557	71		497	106		554	103	
Dp 3 glu	1135	29	a	1306	310	a	1075	96	a	665	164	b	579	37	b
Pt 3 glu	24	5	a	28	1	a	25	6	a	15	2	b	18	2	b
Pl 3 glu	0.49	0.48		0.16	0.28		0.16	0.28		0.49	0.49		0.49	0.01	
Mv 3 glu	47	1		59	16		69	21		41	14		57	16	
Pn 3 glu	11	1		10	3		13	3		12	2		19	4	
Cy 3 gal	214	44		250	48		248	44		212	44		197	17	
Dp 3 gal	95	9	a	100	5	a	108	17	a	61	12	b	47	3	b
Pt 3 gal	31	5	b	42	4	a	43	5	a	25	6	b	22	1	b
Pl 3 gal	0.05	0.04		0.12	0.11		0.09	0.08		0.10	0.02		0.19	0.08	
Mv 3 gal	14	2	b	31	10	a	33	16	a	16	4	b	20	5	a,b
Pn 3 gal	39	12		55	36		60	29		53	17		72	7	
Cy 3 ara	159	42		193	35		152	39		162	16		145	27	
Dp 3 ara	152	15	a	167	16	a	159	14	a	90	18	b	70	12	b
Pt 3 ara	43	7	a,b	64	10	a	57	10	a	35	6	b	32	11	b
Mv 3 ara	23	4	b	48	15	a	46	17	a	25	2	b	43	15	a
Pn 3 ara	11	3		16	8		18	12		13	5		17	8	

glu = glucose, gal = galactose, ara = arabinose, coum = coumaroyl, Av. = average of three replicates, SD = standard deviation, St. = statistics.

The compounds marked with asterisk (**) are first time detected in bilberry fruits to present. Significant differences by Tukey HSD (P < 0.05) in response to the light treatments are marked by different letters for each compound.

### Effect of monochromatic light on phenolic composition of ripe bilberry fruits

**Figure 1:**
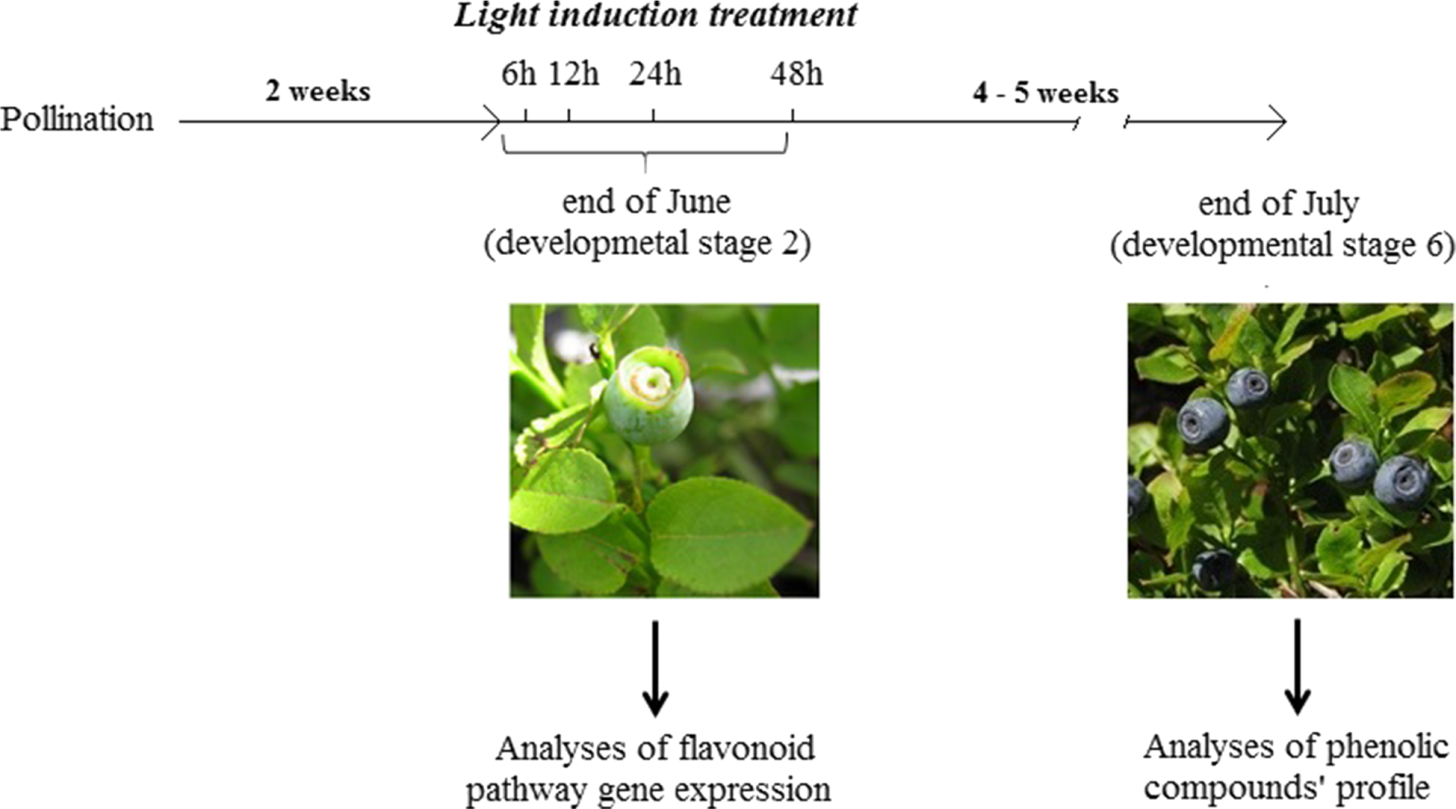
**Design of light treatments and sample collections during the ripening process of bilberry fruits.** Bilberry plants with unripe berries (developmental stage 2, about 2 weeks after pollination) were kept for 14 h in darkness (0 h sample) and then exposed to continuous blue, red, far-red or white light for 48 h. A set of plants left in continuous darkness (dark treatment) for 48 h represented negative control. After the light treatments, plants were grown in greenhouse under natural photoperiod and controlled temperature (21 ± 1°C) until ripening of fruits (developmental stage 6).

In order to investigate the effect of light quality on flavonoid accumulation in ripe berries, bilberry plants were treated with selected wavelengths of the visible light spectrum (blue, red, far-red or white light) during the fruit development process or left in the dark, as detailed in Figure 1. The effect of monochromatic light treatments during berry development on phenolic compounds in ripe berries is shown in Table 1. Significant variations (P < 0.05) were detected in flavonols and proanthocyanidin compounds for some of the light treatments. The level of quercetin 3-*O*-galactose was significantly (P < 0.05) lower in blue light treated plants compared with the other treatments. The levels of myricetin hexoses on the other hand were significantly higher under the red and far-red light treatments. On the contrary, the amounts of procyanidin A2 were lower under red and far-red light treatments, and procyanidin B1 level was higher under white light treatment compared with all the other light treatments.

### Monochromatic light affects anthocyanin composition of ripe bilberry fruits

**Figure 2:**
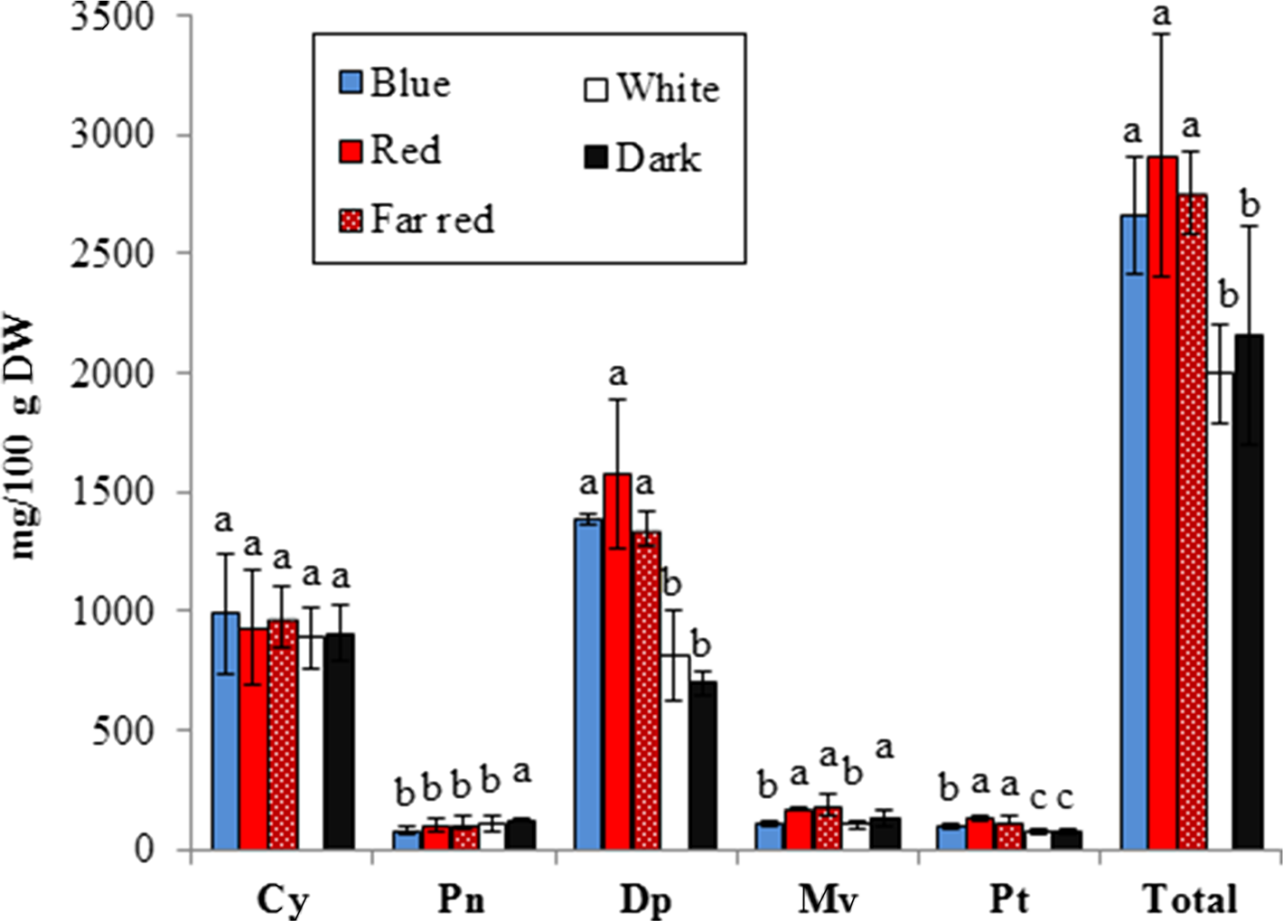
**Concentration of anthocyanidin classes in ripe bilberry fruits treated with different light wavelengths (blue, red, far-red or white) or in dark conditions (n = 3).** Pg’s are not reported here due to their low amounts compared to the other classes of anthocyanidins (Dp, Cy, Pt, Pn, Mv). For each class of anthocyanidin and the total amount of anthocyanins, significant differences by Tukey HSD (P < 0.05) in response to the light treatments are marked by different letters.

The most prominent effect of monochromatic light treatments was seen on anthocyanin content. Figure 2 shows the effect of light treatments on the total amount of each class of anthocyanidins (Dp, Cy, Pn, Mv, Pt, Pg) calculated from the sum of the individual anthocyanin glycosides (Table 2). From the results it is evident that the content of Cy and Pn was not affected by the light treatments, whereas Dp, Mv and Pt showed a significant (P < 0.05) increase (33%, 46% and 38%, respectively) in berries of the plants treated with monochromatic light wavelengths when compared to the berries of the plants grown in white light conditions, suggesting that light quality affects the flavonoid pathway. The content of Mv showed a different behavior than Dp and Pt content; the concentration of Mv was significantly higher (P < 0.05) in berries left in dark than under any of the light treatments (Figure 2). Table 2 shows effect of each of the light treatments on the accumulation of specific anthocyanin compounds. Red and far-red light treatment increased Dp, Mv and Pt compounds conjugated with glucose, galactose and arabinose sugars, but had no effect on the acylated and coumaroylated compounds. The same increase was induced by blue light, with the exception of Pt 3-*O*-galactose, Pt 3-*O*-arabinose, Mv 3-*O*-galactose and Mv 3-*O*-arabinose.

The expression of flavonoid pathway genes *VmCHS*, *VmF3′5′H, VmDFR*, *VmANS* and *VmANR,* and the transcription factor *VmMYB2* were also measured during the monochromatic light treatments at the stage of immature berries. The most of the examined genes showed increase in their expression during the first 12 hours of the study in the plants treated with monochromatic light compared with plants kept in darkness or under white light, even though variation between samples and time points was high (Additional file 2). However, *VmANS* showed the evident increase in the expression after 24 and 48 hours under monochromatic light when compared to dark treated plants. On the contrary, under white light, the expression was not increased compared to dark treated plants. Monochromatic light continued to up-regulate the expression of *VmANS* over dark treated plants throughout the light treatment until 48 h, when the gene was increased up to 3-, 2- and 3.5-folds under blue, red and far-red light treatments, respectively, compared to dark treated plants. Under white light, the expression was only slightly increased (up to 1.3-fold) compared to dark treated plants (Additional file 2).

## Discussion

Recent studies have shown that bilberry populations growing at northern latitudes contain higher amounts of flavonoids, in particular anthocyanins, in comparison to the southern populations [8,9]. The phenomenon is known to be under strong genetic control [9] even though environmental factors may also be involved in the regulation. Solar radiation is one of these factors, and it is known to increase the expression of the flavonoid biosynthesis genes and the content of flavonoids in bilberry leaves [15,31]. Moreover, higher amounts of anthocyanins were found in bilberry fruits grown in controlled conditions in a phytotrone in 24 h natural daylight, mimicking the light conditions of Arctic summers [10]. In the present study, the total anthocyanin content in ripe berries was significantly increased by monochromatic lights of blue, red and far-red, in comparison to fruits treated with white light or kept in darkness (Figure 2). Various effects of monochromatic light wavelengths on anthocyanin biosynthesis have also been reported in other species. For example, in turnip hypocotyls, far-red light had the most prominent effect on anthocyanin biosynthesis, comparable with the amount reached under sunlight [26]. In *Gerbera*, anthocyanin accumulation in flowers was particularly stimulated by blue light [21]. Blue light has been found to significantly increase the biosynthesis of anthocyanins also in fruit species, such as strawberries [25] and grape fruits [22,23], while in cranberry fruits, red and far-red light increased the anthocyanin accumulation over white light [20].

A possible explanation of the present results can be found from the gene expression analyses of flavonoid pathway genes. The expression of the genes *VmCHS, VmF3′5′H*, *VmDFR* and *VmANR* was less influenced by the light treatments (Additional file 2), which was consistent with the detected levels of flavanones, flavonols, stilbenes and proanthocyanidins in the berries kept under different light treatments (Table 1). Moreover, in earlier studies it has been shown that flavonoid pathway genes, for instance *CHS*, can have a diurnal rhythm [33,34]. This is one factor that can have affected the variation in the gene expression results between the different time points. On the contrary, the expression of *VmANS*, which is the key gene in the biosynthesis of anthocyanins, shows a clear increasing trend under monochromatic light treatments, while white light and dark treatment does not have influence. Blue, red and far-red light all up-regulated the expression of *VmANS* already within the first 6 h after the beginning of the light treatment and also throughout the 2-day treatment (Additional file 2). According to Jaakola et al. [7], *VmANS* is expressed only at a very low level in bilberry fruits at the early stage of fruit development. However, the early stages of berry development appeared to be reactive to the light treatments in the present study. Monochromatic light treatments affected the accumulation of anthocyanins by increasing the expression of *VmANS* already at this early stage of berry development.

The higher amount of total anthocyanins in bilberry fruits in response to monochromatic light wavelengths was due to the increased production of Dp’s and Pt’s over Cy’s and Pn’s (Table 2, Figure 2). In the present study, the bilberry plants originated at the 65°N latitude and the amounts of Cy’s and Dp’s produced in plants treated with monochromatic lights were similar to the studies in which berries were grown in natural environment at similar latitudes (64°N [9] and 66°N [10]). Plants kept under white light or in darkness, showed a significant decrease in the content of Dp’s, indicating that the spectral composition of light is involved in the accumulation of this class of anthocyanidins. Considering that in northern latitudes, summer nights are characterized by long twilight with high ratios of blue and far-red light [35], the present study emphasizes that northern light environment promote the accumulation of anthocyanins in bilberry already at the early stages of fruit ripening, by inducing qualitative and quantitative changes in anthocyanin content of ripe fruits.

## Conclusions

We showed that the treatment of bilberry plants under monochromatic light wavelengths of the visible light spectrum, for even short times during the ripening period of the fruits, is enough to induce a significant increase in the anthocyanin content in ripe fruits. Moreover, the quality of light affected particularly the biosynthesis of delphinidin glycosides. Our results indicate that the spectral composition of light regulates the accumulation of anthocyanins in fruits, showing an interaction between the flavonoid biosynthetic pathway and the composition of the light spectrum received by the plant.

## Methods

### Plant material

Bilberry (*Vaccinium myrtillus* L.) plants were harvested from three different locations I-III (I: 65° 06′ N, 25° 5′ E; II: 65° 04′ N, 25° 31′ E; III: 65° 03′ N, 25° 28′ E) in forest stands in Finland. Plants were collected, in each location, within an area of 10 m x 10 m, assuming that the plants within this area belonged to the same genetic background [36] and thus represented specific ecotypes. Plants were collected at the stage when their fruits were small and green, presenting developmental stage 2 (Figure 1). Plants were harvested with their root system and were placed in boxes (50 cm × 70 cm) containing forest peat soil.

After pollination, berries take usually six to seven weeks to ripe in natural stands of Finland. Bilberry fruit ripening stages were identified according to Jaakola et al. [6] and are presented in Figure 1. Developmental stage 2 represented small green unripe berries of 3 to 4 mm in size, approximately two weeks after pollination (end of June). At ripeness (developmental stage 6), which occurs about six weeks after pollination (end of July), the berries were 6 to 8 mm in diameter and turned to dark blue.

### Light sources

**Figure 3:**
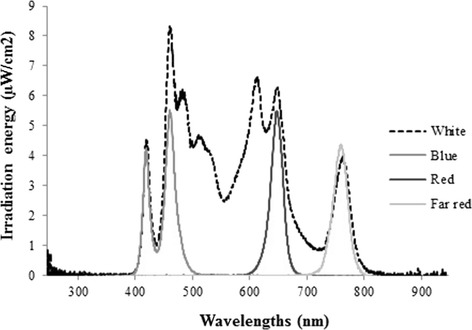
**Light spectra used for the 48 h light treatment experiments in bilberry plant.** White, 400–800 nm; blue, 400–500 nm; red, 600–700 nm; and far-red, 700–800 nm.

Selador led lamps by PALETTA™ (BMI supply, Queensbury, NY, USA) were used to irradiate plants with blue (400–500 nm), red (600–700 nm), far-red (700–800 nm) and white light (400–800 nm, Figure 3) wavelengths. The plants irradiated under blue light received a photon fluence rate of 8.10 μmol m^−2^ s^−1^, under red 7.8 μmol m^−2^ s^−1^, under far-red 7.6 μmol m^−2^ s^−1^ and under white 43.04 μmol m^−2^ s^−1^. Plants exposed to white light were considered as a positive control. A set of plants kept in total darkness was considered as negative control. Light measurements were conducted by using USB RAD+ spectroradiometer (Ocean Optics Inc., Dunedin, FL, USA).

### Light treatments and sample collection

Bilberry plants were treated with each specific light wavelength during the berry ripening period, as shown in Figure 1. Pools of bilberry plants from each location (I-III), were used for the treatments. Plants holding berries at stage 2, were initially kept in darkness for 14 h and then exposed to the continuous blue, red, far-red or white light induction or placed to darkness for 48 h (Figure 1). The berry developmental stage 2 was selected for the experiments based on preliminary analyses (data not shown) which indicated stage 2 to be the most reactive one, among all the bilberry fruit ripening stages, in the expression of flavonoid pathway genes in response to the light illumination. The light treatments were conducted in growth chambers with controlled temperature (21 ± 1°C) and humidity (60%) to erase the effect of temperature on flavonoid biosynthesis. After the light treatment, growth of plants was conducted in greenhouse under controlled temperature condition (21 ± 1°C) and natural photoperiod. When fully ripened (stage 6, Figure 1), the berries were harvested and stored at −80°C before freeze-dried within six months. The light treatments did not affect the process of ripening of the berries. Freeze-dried berries were stored in a desiccator at −20°C until analysed for metabolic compounds.

### Metabolic analyses

The ground material (100 mg out of 3 g) of each sample was extracted with 1.5 mL of 80% methanol on shaking for 1 h. Samples were centrifuged at 12000 g for 2 min (Sigma 3-30 k, Osterode, Germany) and the supernatants were collected. The extraction was repeated and the supernatants were combined and brought to a volume of 5 mL. After filtering (0.22 μm PVDF filters) and transferring to glass vials, the samples were randomized and analyzed for anthocyanins, flavonols, proanthocyanidins, stilbenes and other phenolic compounds by UPLC-MS/MS.

### Analysis of phenolic compounds

Flavonols, flavanones, hydroxycinnamic acids, proanthocyanidins and stilbenes were analysed as described in Vrhovsek et al. [28]. Chromatography, mass spectrometry conditions and multiple reaction monitoring (MRM) transitions can be found in the referred literature. Quantification was made by external calibration curves, injecting authentic standards of each of the detected compounds at different concentrations.

### Analysis of anthocyanins

Anthocyanins were analysed by using UPLC-MS/MS as described by Arapitsas et al. [30]. Anthocyanins were detected by MRM, by screening the MS/MS transitions and using the parameters described in Additional file 3. For some of the compounds, there were no standards available, but they could be tentatively identified on the basis of their MRM transitions and the relative retention time, in respect to known compounds and considering previous results [37]. For example, standards of the galactoside derivatives of cyanidin and peonidin were available, and these compounds seem to elute before but closely to the respective glucoside derivatives (peaks 1, 2 and 22, 23 in Additional file 3). As such, the peak eluting 0.15 seconds before malvidin glucoside showing the same MRM transition is likely to be malvidin galactose (peak 15 in Additional file 3), and this reasoning can also be applied to the other galactoside and arabinoside derivatives.

For quantification, external calibration curves were prepared by injecting authentic standards of each compound at different concentrations. In case the authentic standard was not available, the anthocyanins were quantified relative to malvidin-3-*O*-glucose, using the malvidin-3-*O*-glucose calibration curve (Additional file 3).

### Statistical analysis

The effect of the light treatment on every metabolite analyzed in the berries was tested with One-way ANOVA. Multiple comparisons were made by Tukey HSD’s post-hoc test. The tests were performed using STATISTICA version 12.

### Supplementary analyses

A supplementary study was conducted in order to study if the increased amount of anthocyanins was related to the gene expression of flavonoid pathway genes in bilberries (Additional file 1). Bilberry plants from locations I and II, with berries at developmental stage 2, were initially kept in darkness for 14 h (0 h sample) and then exposed to the continuous blue, red, far-red or white light induction or placed to darkness for 48 h. During the light treatment, berry samples were collected for RNA isolation after 0, 6, 12, 24 and 48 h of treatment. Samples were immediately stored at −80°C until analysed for gene expression.

### Isolation of RNA and cDNA preparation

Total RNA was isolated from bilberry fruits at stage 2 that were collected after 0, 6, 12, 24 and 48 h from the beginning of the light treatments. The RNA was isolated according to the method of Jaakola et al. [38] with the exception that the phenol-chloroform extraction was substituted with the RNA purification protocol in E.Z.N.A.® Total RNA Kit I (Omega Bio-Tek, Norcross, GA, USA). The quality of the isolated RNA was verified by measuring the absorbance spectrum with NanoDrop N-1000 spectrophotometer (NanoDrop Technologies, Thermo Scientific, Wilmington, DE, USA) and on a 1% (w/v) ethidium bromide-stained agarose gel. RNA was converted to cDNA with RevertAid Premium Reverse Transcriptase (Thermo Scientific) in accordance with the manufacturer’s instruction. RNA extraction (and further gene expression analyses) was repeated twice for each set of plants.

### Gene expression analysis

Transcript accumulation of the genes *VmCHS*, *VmF3′5′H*, *VmDFR*, *VmANS* and *VmANR*, and the transcription factor *VmMYB2* was detected using the LightCycler SYBR Green qPCR Kit (Roche Applied Sciences, Indianapolis, IN, USA). The primers used for the amplification are listed in Additional file 4.

Analyses with qPCR were performed with a LightCycler 2.0 instrument and software (Roche). The PCR conditions were 95°C for 10 min, followed by 45 cycles of 95°C for 10 s, 60°C for 20 s, and 72°C for 10 s. *VmACT* gene (Additional file 4) was used as a reference gene for relative quantification. Differential gene expression levels were calculated by comparing each of treatments to treatment 0 h.

## Additional files

Additional file 1:
**The flavonoid biosynthetic pathway of bilberry with particular emphasis on anthocyanin classes.** Enzymes for each step are shown in capitals. Enzymes required for flavonoid synthesis; *PAL*, phenylalanine ammonia-lyase; *C4H*, cinnamate 4-hydroxylase; *4CL*, 4-coumaroyl:CoA ligase; *CHS*, chalcone synthase; *CHI*, chalcone isomerase; *F3H*, flavanone 3′-hydroxylase; *F3′* *H*, flavonoid 3′-hydroxylase; *F3′5′* *H*, flavonoid 3′,5′-hydroxylase; *FLS*, flavonol synthase; *DFR*, dihydroflavonol 4-reductase; *ANS*, anthocyanidin synthase; *ANR*, anthocyanidin reductase; *UFGT*, UDP glucose-flavonoid 3-*O*-glucosyl transferase; *MT*, methyltransferase. The transcript levels of the genes *CHS*, *F3′5′* *H*, *DFR*, *ANS* and *ANR* (in the figure marked with a square) was analyzed in response to the exposure to different light wavelengths. Additional file 2:
**Relative transcript abundance of the flavonoid pathway genes** ***VmCHS,*** ***VmF3′*** ***5***′***H,*** ***VmDFR,*** ***VmANS*** **and** ***VmANR,*** **and the transcription factor** ***VmMYB2*** **in bilberry fruits (at stage 2) after 6, 12, 24 and 48 h under different light conditions.** Data represent average and SD values of samples collected from two locations (see Methods). Additional file 3:
**UPLC-MS/MS data for anthocyanin quantification.** In case of two MRM transitions for a given compound, the first was used as quantifier and the second as qualifier. RT = retention time, CV = cone voltage, CE = collision energy, Std = standard curve. Additional file 4:
**Sequences of the primers used in qPCR to determine gene transcripts.**

## Abbreviations

UPLC-MS/MS: Ultra performance liquid chromatography – tandem mass spectrometer
Dp: Delphinidin
Cy: Cyanidin
Pt: Petunidin
Pn: Peonidin
Mv: Malvidin
Pg: Pelargonidin
Glu: Glucose
Gal: Galactose
Ara: Arabinose
Coum: Coumaroyl
DW: Dry weight
*VmCHS*: Vaccinium myrtillus chalcone synthase
*VmF3′5′**H*: Vaccinium myrtillus flavonoid 3′5′-hydroxylase
*VmDFR*: Vaccinium myrtillus dihydroflavonol 4-reductase
*VmANS*: Vaccinium myrtillus anthocyanidin synthase
*VmANR*: Vaccinium myrtillus anthocyanidin reductase
*VmMYB2*: Vaccinium myrtillus MYB2 transcription factor
*VmACT*: Vaccinium myrtillus actin
MRM: Multiple reaction monitoring
