# Supplementary figures and images for: Monochromatic light increases anthocyanin content during fruit development in bilberry
Source: BMC Plant Biol. 2014 Dec 16;14:377. doi: 10.1186/s12870-014-0377-1 (PMC4274681; doi:10.1186/s12870-014-0377-1)

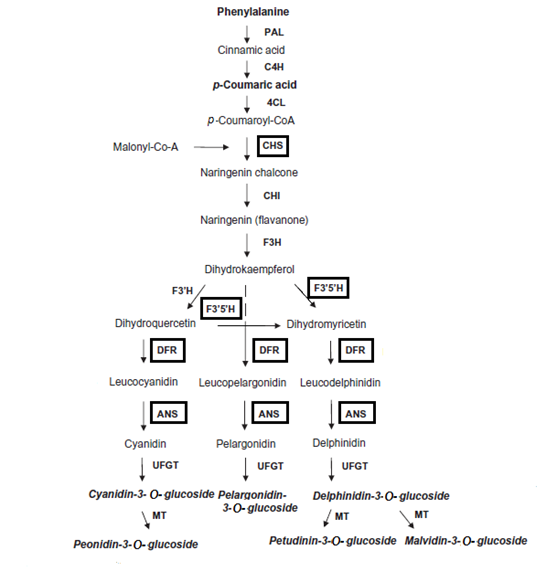

Supplement: Additional file 1: — The flavonoid biosynthetic pathway of bilberry with particular emphasis on anthocyanin classes. Enzymes for each step are shown in capitals. Enzymes required for flavonoid synthesis; PAL, phenylalanine ammonia-lyase; C4H, cinnamate 4-hydroxylase; 4CL, 4-coumaroyl:CoA ligase; CHS, chalcone synthase; CHI, chalcone isomerase; F3H, flavanone 3′-hydroxylase; F3′ H, flavonoid 3′-hydroxylase; F3′5′ H, flavonoid 3′,5′-hydroxylase; FLS, flavonol synthase; DFR, dihydroflavonol 4-reductase; ANS, anthocyanidin synthase; ANR, anthocyanidin reductase; UFGT, UDP glucose-flavonoid 3-O-glucosyl transferase; MT, methyltransferase. The transcript levels of the genes CHS, F3′5′ H, DFR, ANS and ANR (in the figure marked with a square) was analyzed in response to the exposure to different light wavelengths. [file 12870_2014_377_MOESM1_ESM.png]

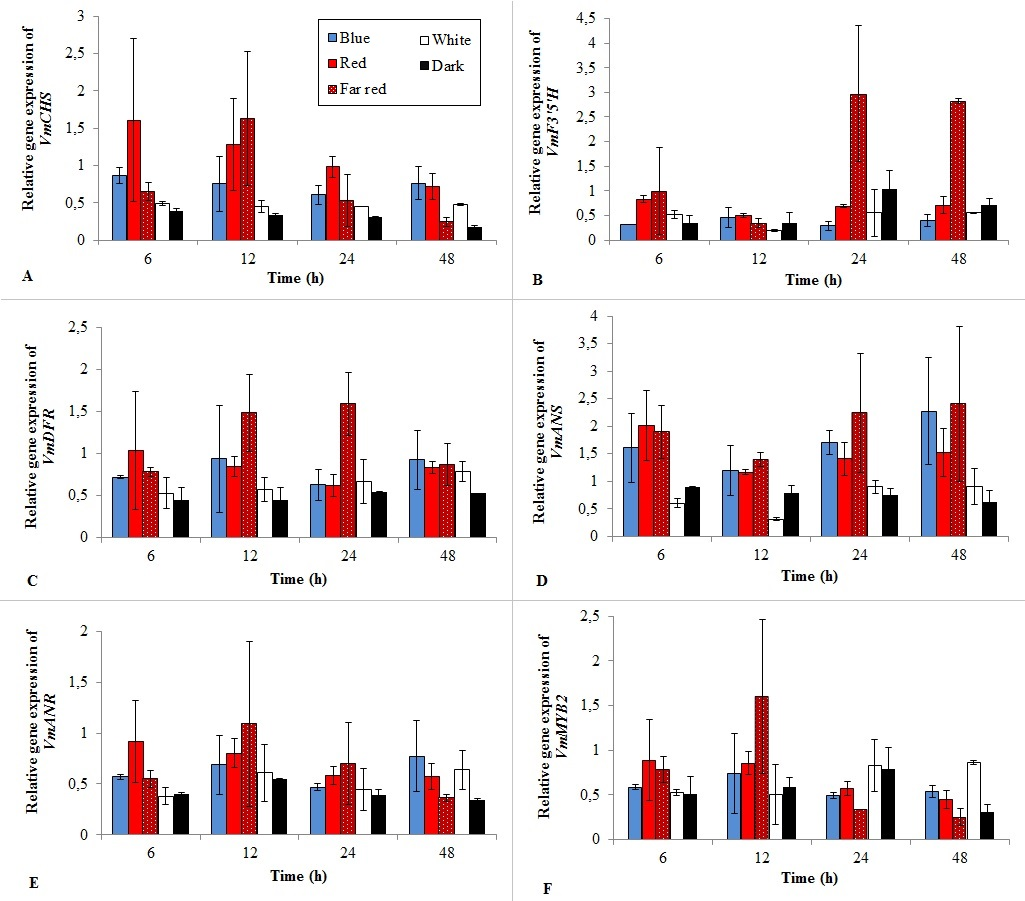

Supplement: Additional file 2: — Relative transcript abundance of the flavonoid pathway genes VmCHS, VmF3′ 5′H, VmDFR, VmANS and VmANR, and the transcription factor VmMYB2 in bilberry fruits (at stage 2) after 6, 12, 24 and 48 h under different light conditions. Data represent average and SD values of samples collected from two locations (see Methods). [file 12870_2014_377_MOESM2_ESM.png]
