# Supplementary material for: Monochromatic light increases anthocyanin content during fruit development in bilberry
Source: BMC Plant Biol. 2014 Dec 16;14:377. doi: 10.1186/s12870-014-0377-1 (PMC4274681; doi:10.1186/s12870-014-0377-1)
Supplement: Additional file 3: — UPLC-MS/MS data for anthocyanin quantification. In case of two MRM transitions for a given compound, the first was used as quantifier and the second as qualifier. RT = retention time, CV = cone voltage, CE = collision energy, Std = standard curve. [file 12870_2014_377_MOESM3_ESM.doc]

**Additional file 3 UPLC-MS/MS data for anthocyanin quantification.** In case of two MRM transitions for a given compound, the first was used as quantifier and the second as qualifier.

| **Peak** | **Identification** | **RT** | **MRM transitions** | **CV** | **CE** | **Quantified by** |
| --- | --- | --- | --- | --- | --- | --- |
| 1 | Cy 3 gal | 3.03 | 449 → 287; 136 | 28 | 30; 48 | Std |
| 2 | Cy 3 glu | 3.19 | 449 → 287; 136 | 28 | 30; 48 | Std |
| 3 | Cy 3 ara | 3.36 | 419 → 287; 137 | 26 | 24; 52 | Std |
| 4 | Cy coum 3 gal | 4.89 | 595 → 287; 137 | 34 | 34; 72 | Mv 3 glu |
| 5 | Cy coum 3 glu | 5.14 | 595 → 287; 137 | 34 | 34; 72 | Std |
| 6 | Cy acetyl 3 gal | 4.05 | 491 → 287; 213 | 28 | 28; 54 | Mv 3 glu |
| 7 | Cy acetyl 3 glu | 4.44 | 491 → 287; 213 | 28 | 28; 54 | Mv 3 glu |
| 8 | Cy 3 sambubioside | 3.15 | 581 → 287; 137 | 30 | 28; 66 | Mv 3 glu |
| 9 | Dp 3 gal | 2.64 | 465 → 303; 229 | 20 | 22; 58 | Mv 3 glu |
| 10 | Dp 3 glu | 2.82 | 465 → 303; 229 | 20 | 22; 58 | Std |
| 11 | Dp 3 ara | 2.90 | 435 → 303 | 20 | 22 | Mv 3 glu |
| 12 | Dp coum 3 gal | 4.57 | 611 → 303; 229 | 34 | 28; 70 | Mv 3 glu |
| 13 | Dp coum 3 glu | 4.83 | 611 → 303; 229 | 34 | 28; 70 | Std |
| 14 | Dp acetyl 3 glu | 4.38 | 507 → 303; 229 | 30 | 30; 50 | Mv 3 glu |
| 15 | Mv 3 gal | 3.74 | 493 → 331; 315 | 28 | 24; 34 | Mv 3 glu |
| 16 | Mv 3 glu | 3.89 | 493 → 331; 315 | 28 | 24; 34 | Std |
| 17 | Mv 3 ara | 4.00 | 463 → 331 | 28 | 24 | Mv 3 glu |
| 18 | Mv coum 3 gal | 5.33 | 639 → 331; 315 | 38 | 30; 58 | Mv 3 glu |
| 19 | Mv coum 3 glu | 5.58 | 639 → 331; 315 | 38 | 30; 58 | Std |
| 20 | Mv acetyl 3 gal | 4.67 | 535 → 331; 315 | 30 | 26; 50 | Mv 3 glu |
| 21 | Mv acetyl 3 glu | 5.01 | 535 → 331; 315 | 30 | 26; 50 | Std |
| 22 | Pn 3 gal | 3.59 | 463 → 301; 286 | 28 | 28; 42 | Std |
| 23 | Pn 3 glu | 3.76 | 463 → 301; 286 | 28 | 28; 42 | Std |
| 24 | Pn 3 ara | 3.93 | 433 → 301; 286 | 26 | 22; 40 | Mv 3 glu |
| 25 | Pn coum 3 gal | 5.29 | 609 → 301; 286 | 38 | 32; 54 | Mv 3 glu |
| 26 | Pn coum 3 glu | 5.57 | 609 → 301; 286 | 38 | 32; 54 | Std |
| 27 | Pn acetyl 3 glu | 4.94 | 505 → 301; 286 | 30 | 28; 50 | Mv 3 glu |
| 28 | Pt 3 gal | 3.24 | 479 → 317; 302 | 28 | 30; 42 | Mv 3 glu |
| 29 | Pt 3 glu | 3.39 | 479 → 317; 302 | 28 | 30; 42 | Std |
| 30 | Pt 3 ara | 3.50 | 449 → 317 | 28 | 30 | Mv 3 glu |
| 31 | Pt acetyl 3 glu | 4.88 | 521 → 317; 302 | 28 | 24; 46 | Mv 3 glu |
| 32 | Pg 3 gal | 3.36 | 433 → 271; 121 | 24 | 36; 58 | Mv 3 glu |
| 33 | Pg 3 glu | 3.51 | 433 → 271; 121 | 24 | 36; 58 | Std |

RT = retention time, CV = cone voltage, CE = collision energy , Std = standard curve
