# Supplementary material for: Monochromatic light increases anthocyanin content during fruit development in bilberry
Source: BMC Plant Biol. 2014 Dec 16;14:377. doi: 10.1186/s12870-014-0377-1 (PMC4274681; doi:10.1186/s12870-014-0377-1)
Supplement: Additional file 4: — Sequences of the primers used in qPCR to determine gene transcripts. [file 12870_2014_377_MOESM4_ESM.doc]

**Additional file 4 Sequences of the primers used in qPCR to determine gene transcripts.**

| **Gene** | **Sequence of forward primer** | **Sequence of reverse primer** |
| --- | --- | --- |
| *VmCHS* | 5′-CCAAGGCCATCAAGGAATG-3′ | 5′-TGATACATCATGAGTCGCTTCAC-3′ |
| *VmF3′5′H* | 5′-AAGCGTATGCATAGCAAGTGG-3′ | 5′-TGATTCGGTGCCTTGAGAAT-3′ |
| *VmDFR* | 5′-GAAGTGATCAAGCCGACGAT-3′ | 5′-ATCCAAGTCGCTCCAGTTGT-3′ |
| *VmANS* | 5′-TCTTCTACGAGGGCAAATGG-3′ | 5′-ACAGCCCATGAAATCCTGAC-3′ |
| *VmANR* | 5′-GCTGGTGTTTCTCCCACAAT-3′ | 5′-AAATATATGGGCGCGACAAA-3′ |
| *VmMYB2* | 5′-ATTCAACTTCATGGCGAAGG-3′ | 5′-GGCATGCATTCTTATAATGAGGT-3′ |
| *VmACT* | 5´-TTCCCTGGGATTGCTGATAG-3´ | 5´-GGTCTTGGCAATCCACATCT-3´ |
